# 

**Published:** 1863-11

**Authors:** 


### CONTENTS.

ORIGINAL ESSAYS AND COMMUNICATIONS.

Anæsthetics. By J. D. White, D. D. S. .......... 465
Absorption of Fangs of the Temporary Teeth. By Wm. H. Atkinson, M. D. .......... 473
Plugging Teeth with Gold. By B. Wood, M. D. .......... 476

PROCEEDINGS OF SOCIETIES.

First Annual Meeting of the Dental Association of Western New York.......... 483

SELECTIONS.

Iodide of Potassium and Carb, Potash in Rheumatism.......... 491
Crust of Bread.......... 493
Power of the Imagination.......... 493
Chloride of Iron and Chloride of Sodium as a Hæmostatic .......... 494
Electrical Phenomena in the Alps.......... 495
Sugar as an Anthelmintic.......... 495
Necrosis of Lower Jaw.......... 495
Fermentation as a Cause of Various Diseases.......... 496
Physiological effects of Exercise upon the Human Body.......... 499
Sulphur rendered soft.......... 501
Test for Olive Oil.......... 502
Hair-Lip and Cleft Palate.......... 502
Hygienic Importance of Light.......... 503
Reform in Medical Education.......... 503
Iowa State Dental Society.......... 507

EDITORIAL.

Western New York Dental Society.......... 507
Transactions of the American Dental Society.......... 508
Pittsburgh Dental Society.......... 509
Nitrous Oxide as an Anæsthetic .......... 510

